# Nurse-Led Strategies for Lifestyle Modification to Control Hypertension in Older Adults: A Scoping Review

**DOI:** 10.3390/nursrep15030106

**Published:** 2025-03-18

**Authors:** Shuangshuang Li, Stephanie Craig, Gary Mitchell, Donna Fitzsimons, Laura Creighton, Gareth Thompson, Patrick Stark

**Affiliations:** School of Nursing and Midwifery, Queen’s University Belfast, Belfast BT9 7BL, UK; sli49@qub.ac.uk (S.L.); s.craig@qub.ac.uk (S.C.); gary.mitchell@qub.ac.uk (G.M.); d.fitzsimons@qub.ac.uk (D.F.); laura.creighton@qub.ac.uk (L.C.); gareth.thompson@qub.ac.uk (G.T.)

**Keywords:** hypertension, older people, blood pressure, non-pharmacological interventions, health, interventions, scoping review

## Abstract

High blood pressure in older adults poses significant risks, including cardiovascular disease, stroke, and renal failure; yet, its management is often overlooked. Nurse-led personalised interventions provide essential guidance, helping patients adhere to treatment plans and adopt lifestyle changes, improving outcomes and quality of life. A scoping review of the literature was conducted following the Preferred Reporting Items for Systematic Reviews and Meta-Analysis Extension for Scoping Reviews (PRISMA-ScR). Six electronic databases were searched systematically (CINAHL, MEDLINE, PsycINFO, EMBASE, Web of Science, and Scopus). Five research studies were included in this review, from five countries (India, Korea, China, Turkey and Thailand). Primary data were synthesised using descriptive and thematic analysis methodology. The five main themes from this review relate to nurse-led empowerment strategies for hypertension management, variability in blood pressure outcomes, the importance of tailored education and counselling, the role of regular follow-ups and support, and environmental support. Overall, nurse-led personalised interventions improve blood pressure management and patient engagement in older adults, highlighting the need for research into their long-term effectiveness and broader applicability.

## 1. Introduction

Hypertension (HTN) or high blood pressure (BP) represents a major public health challenge worldwide, particularly among older adults [1], where its prevalence and impact are both profound and multifaceted. Defined as elevated BP levels [2], HTN is associated with significant risks, including cardiovascular disease, stroke, cognitive decline, and renal dysfunction [3]. Recent global estimates from the World Health Organisation [4] highlight that over 1.28 billion adults live with HTN, with many remaining untreated or inadequately managed. Due to age-related physiological changes and comorbidities, older people bear a disproportionate amount of this global burden and are especially vulnerable to the negative effects of high BP [5]. Despite advances in pharmacological therapies and non-pharmacological interventions [6], managing HTN in this population remains a pressing and complex issue requiring a multidimensional approach [7].

The evolution of HTN diagnostic criteria, as seen in the transition from the Joint National Committee’s (JNC 7) [8] recommendations to the 2017 American College of Cardiology (ACC) [9] and American Heart Association (AHA) [9] guidelines, highlights the heightened urgency to manage BP more stringently in older adults [10]. Although the 2017 AHA guidelines [9] primarily address individuals aged 65 and above, various organisations, such as the American Academy of Family Physicians (AFP), advocate for managing HTN starting at age 60 [11]. The guidelines recommend maintaining systolic BP at approximately 130 mm Hg for older adults, reflecting a growing recognition of the relationship between elevated BP and adverse health outcomes [11]. However, older adults often face unique challenges in achieving optimal BP control [12], including frailty, polypharmacy, and the U-shaped relationship between systolic BP and mortality, where both excessively high and low BP levels are associated with increased mortality risks [1].

Pharmacological management remains the cornerstone of HTN treatment, with medications such as diuretics, calcium channel blockers, and angiotensin-converting enzyme (ACE) inhibitors forming the backbone of therapy [13]. However, these therapies are not without limitations. Older adults often experience age-related changes in renal and cardiovascular function [3], necessitating careful dose adjustments to mitigate the risk of adverse effects. Furthermore, the presence of orthostatic hypotension (OH) and frailty further complicate the safe and effective use of antihypertensive medications [7]. These challenges highlight the need for individualised treatment approaches that balance BP reduction with the prevention of treatment-associated risks [14].

Non-pharmacological interventions, including dietary modifications, physical activity, and lifestyle changes, play a crucial role in HTN management, particularly in resource-limited settings where access to medications may be constrained [6]. Evidence supports the efficacy of strategies such as the Dietary Approaches to Stop Hypertension (DASH) [15] diet and structured exercise programs in lowering BP among older adults [16]. However, sustaining these lifestyle changes poses significant challenges, particularly in older populations who may require tailored support and education [17].

The role of nurses in HTN management is increasingly recognised as critical [18], particularly in implementing community-based, patient-centred interventions. Nurse-led initiatives have demonstrated promise in enhancing self-efficacy, improving BP control, and fostering adherence to both pharmacological and non-pharmacological treatment regimens [18]. Nurses’ ability to provide education, counselling, and consistent monitoring makes them uniquely positioned to address the multifactorial challenges of HTN management in older adults [19]. However, the variability in the design and outcomes of existing nurse-led programs shows the need for further research to identify best practices and optimise implementation.

This scoping review aims to address the pressing need for improved HTN management strategies tailored to older adults aged 60 and above. It provides a comprehensive review of the current state of pharmacological and non-pharmacological interventions, highlighting their limitations and potential. Particular emphasis is placed on nurse-led interventions, examining their effectiveness and barriers to implementation. By synthesising existing evidence, this study seeks to inform the development of holistic and scalable approaches to HTN management that enhance the quality of care and health outcomes for older adults.

## 2. Materials and Methods

### 2.1. Design

This scoping review employed a systematic approach, following the methodology guidelines proposed by Arksey and O’Malley [20] and Joanna Briggs Institute (JBI) [21], and guided by the Preferred Reporting Items for Systematic Reviews and Meta-analysis extension for scoping reviews (PRISMA-ScR) checklist [22]. This process involved establishing a research question, identifying relevant studies through the development of eligibility criteria, and selecting relevant studies. Data from the relevant studies were then extracted and analysed, and the results summarised and reported.

### 2.2. Search Strategy

The search strategy was developed with the review team including input from the subject librarian. A preliminary search was also completed using Google Scholar. The Population, Exposure, Outcome (PEO) Framework was used to aid the development of the research question and in devising eligibility criteria for relevant studies [23,24]. Therefore, to address the question of what nurse-led strategies for lifestyle modifications for older people to control blood pressure, the search terms are related to (P) older people; (E) nurse-led and lifestyle modification; and (O) changes in blood pressure.

Six electronic databases—CINAHL, MEDLINE, PsycINFO, EMBASE, Web of Science, and Scopus—were searched over a period of five months, from March 2024 to July 2024. The search terms listed in Table 1 were used, with MeSh terms also used where available in each of the databases.

### 2.3. Eligibility Criteria

No restrictions were placed on the date of publication. Qualitative studies and systematic reviews were excluded. While qualitative studies offer valuable insights into patient experiences, this review focuses on the quantitative impacts of empowerment strategies. Systematic reviews were excluded as synthesised evidence may not include the required level of detail to address the aims of this study, in terms of describing the qualities of interventions or evidence of promise. Systematic reviews could be scanned for relevant references. While this scoping review only includes studies written in English, no geographical restrictions were applied. The included population were older people aged over 60. Table 2 presents full details of the inclusion and exclusion criteria used in this scoping review.

### 2.4. Screening and Extraction

First-level screening included only the title and abstract only. Covidence software (https://www.covidence.org/) [25] was used to aid in the removal of duplicates, screening of literature, and data extraction. Non-English-language papers were also screened out at this first stage. Full-text articles were then reviewed to assess eligibility. Although not mandatory for scoping reviews [22], quality appraisal of the included studies was conducted to provide readers with an understanding of the quality of the evidence. The JBI checklist for quasi-experimental studies [26] was used for quality appraisal of the included studies.

### 2.5. Data Analysis

Data were analysed using thematic analysis, a robust method to identify, analyse, and interpret patterns within data [27]. This involved a six-step framework including familiarisation with the data, generation of initial codes, searching for themes, reviewing themes, defining themes, and, lastly, reporting themes [28]. This process was led by SL, with re-checking, support, and guidance provided by the review team.

## 3. Results

**Figure 1:**
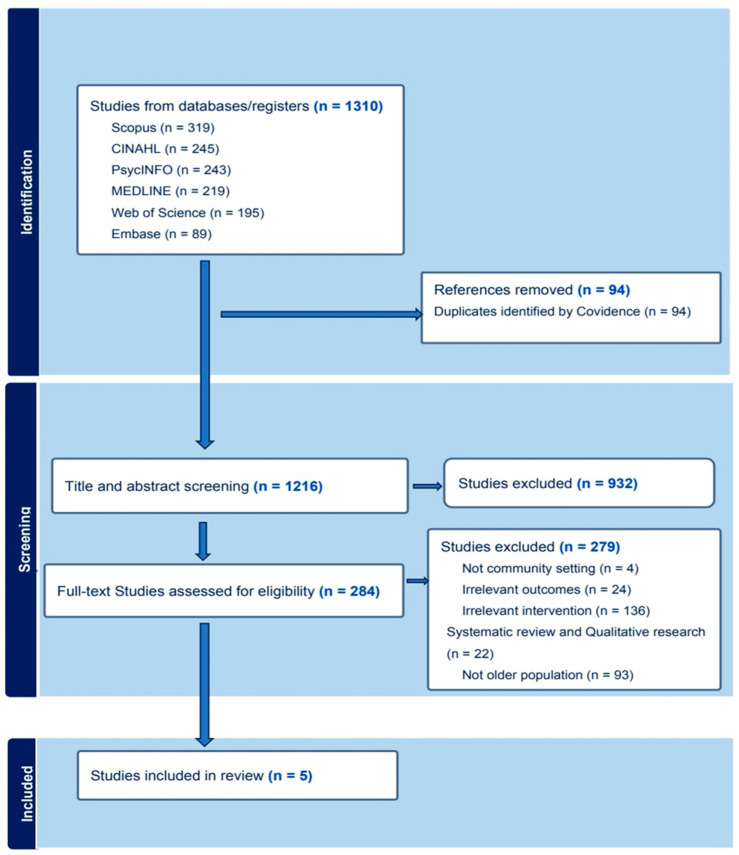
PRIMSA-ScR flowchart.

A total of 1310 studies were initially imported for screening. After removing 94 duplicates, 1216 studies proceeded to title and abstract screening. Of these, 932 were deemed irrelevant and excluded. We sought to retrieve full texts for the remaining 284 studies, which were subsequently screened. Following this process, 5 studies met the inclusion criteria and were included in the review as illustrated in the PRISMA-ScR flowchart in Figure 1.

### 3.1. Characteristics of Included Studies

Five papers met the inclusion criteria. All five studies used a quantitative approach, three studies were full randomised control trials (RCTs), one study was a single-blind RCT, and one study was a quasi-experimental study. Table 3 provides the characteristics of the studies included in this review.

### 3.2. Quality Appraisal

The quality of the literature included in this scoping review was appraised using the CASP tool [33] and JBI [34] critical appraisal checklist for quasi-experimental and RCT studies. Scoring revealed an average of 9.75 for the RCTs and a perfect 9 for the quasi-experimental study, highlighting an overall adequate quality but with variability due to differing study designs. Details of the quality appraisal of each study are included in Table 4 below and individual appraisal forms are available upon request.

### 3.3. Synthesis of Evidence

Nurse-led empowerment strategies were identified through a descriptive analysis of intervention studies. Included in the analysis were study design, characteristics of participants, inclusion or exclusion criteria, nurse-led empowerment strategies, outcomes, measurement instruments used, and key findings. A thematic analysis was then conducted to identify the factors that facilitate nurse-led empowerment strategies in community settings. Themes were generated by analysing the extracted data for patterns across the dataset, in relation to factors that facilitate nurse-led empowerment strategies in community settings. The presentation of themes is in alignment with methodological recommendations from Arksey and O’Malley [20] and Levac et al. [35]. The analysis uncovered five principal themes: (1) Nurse-Led Empowerment Strategies for HTN Management; (2) Variability in Blood Pressure Outcomes; (3) The Importance of Tailored Education and Counselling; (4) the Role of Regular Follow-Ups and Support; and (5) Environmental Support.

#### 3.3.1. Theme 1: Nurse-Led Empowerment Strategies for Hypertension Management

Theme one highlights the efficacy of nurse-led interventions for managing HTN in older adults. Sheilini et al. [29] conducted an RCT in India over six months to evaluate a nurse-led HTN management program. Among 160 participants, 124 completed the study, with 64 receiving interventions. The intervention program included personalised education, informational leaflets, medication reminders, and follow-up calls. Attrition due to family obligations, relocation, and communication issues was noted.

Kim and Park [30] tested auricular acupressure’s effects on blood pressure, stress, and sleep in elderly Koreans through an 8-week single-blind RCT involving 46 participants aged 65–85 years. The 8-week intervention focused on specific acupoints related to HTN, as well as stress reduction, and included the upper triangular fossa, Shenmen, Kidney Yu, Heart Yu, and occipital area, while random points were used for the control group. Limitations included a small sample size, short duration, and potential researcher bias.

Furthermore, Tu et al. [31] investigated a combined intervention approach in China involving education, lifestyle modification, and healthcare consultations in 270 older adults over six months. While the study demonstrated some benefits, the short follow-up period and self-selected sampling limited its generalizability. Kolcu and Ergun [19] assessed a 20-week nurse-led intervention in Turkey among 74 participants. The program integrated six health education sessions on medication use, motivational meetings, and dietary changes, whereas the control group received standard care. Baseline differences and limited generalizability due to the study’s design were noted as limitations.

Sukpattanasrikul et al. [32] implemented a 16-week self-management model in Thailand to test a self-management model for uncontrolled HTN in the older population among 156 participants. Specifically, the intervention was tailored to involve personalised education, guidance in lifestyle modification, regular home visits, and telephonic follow-ups for the intervention group, while the control group received standard care. Overall, these studies highlight the potential of nurse-led HTN management strategies while emphasising the need for larger-scale, long-term, and globally applicable research.

#### 3.3.2. Theme 2: Variability in Blood Pressure Outcomes

The variability in BP outcomes across studies can be attributed to the diverse methodologies employed in interventions. This diversity highlights the importance of tailoring strategies for effective HTN management. Sukpattanasrikul et al. [32] demonstrated a significant reduction in systolic blood pressure (SBP) by 16.32 mm Hg using a quasi-experimental design, contrasting with Sheilini et al. [29], whose randomised controlled trial (RCT) showed only a minor SBP reduction of 1.06 mm Hg. The differences may emerge from intervention design; Sukpattanasrikul et al. [32] employed Individual and Family Self-Management, while Sheilini et al. [29] focused on education and reminders.

Multifaceted interventions often achieve superior outcomes in high-risk groups. Tu et al. [31] conducted a six-month RCT integrating hospital-community collaboration, personalised education, and structured follow-up, achieving a 3.8 mm Hg SBP reduction in the intervention group. By contrast, Sheilini et al. [29] observed only a 1.06 mm Hg reduction in a comparable duration, suggesting that comprehensive, context-sensitive strategies outperform simpler approaches.

Further evidence supports the effectiveness of nurse-led and self-management interventions. Kolcu and Ergun [19] achieved a 10.54 mm Hg SBP reduction through a 20-week program in nursing homes. Similarly, Kim and Park [30] reported a 5.31 mm Hg decrease over eight weeks. Longer, culturally sensitive, and more comprehensive interventions yielded greater BP reductions, highlighting their value in HTN management. These findings highlight the need for adaptable, targeted approaches to effectively manage HTN in older adults.

#### 3.3.3. Theme 3: Importance of Tailored Education and Counselling

Tailored education and counselling are integral to community health strategies, offering personalised approaches that enhance the adherence to prescribed interventions. These interventions address the unique needs of target populations, increasing their effectiveness in improving health outcomes. Sheilini et al. [29] demonstrated the efficacy of multimodal interventions, combining personalised teaching and counselling to promote medication adherence and healthy lifestyles. The study revealed that tailored education fosters high knowledge levels, correlating with better adherence rates and laying a foundation for broader application. Nurse-led interventions focusing on individual skill-building and knowledge enhancement were highlighted as particularly impactful. Tu et al. [31] expanded on this by showing the benefits of personalised discharge guidance and self-care education. This approach emphasised patient-specific goals, improving confidence in managing chronic diseases. While requiring ongoing support, the intervention reinforced the importance of sustained patient involvement for favourable long-term outcomes.

Kolcu and Ergun [19] explored tailored education for older adults managing HTN incorporating flexible, motivational group activities. The intervention significantly improved participants’ adherence to blood pressure management, showing the effectiveness of adapting strategies to individual needs and preferences. Similarly, Sukpattanasrikul et al. [32] integrated culturally sensitive education and training into HTN management programs for older adults. Regular sessions empowered participants with self-care strategies, promoting better health management and cultural preservation. These studies collectively affirm the critical role of tailored education and counselling in achieving significant and sustainable health improvements across diverse populations.

#### 3.3.4. Theme 4: Role of Regular Follow-Ups and Support

Regular follow-ups and structured support are pivotal in managing HTN, especially in older populations. Sheilini et al. [29] utilised telephonic reminders at scheduled intervals, which significantly improved medication adherence over six months. This method promoted consistent follow-up attendance and sustained patient engagement, though the variability in patient responsiveness was not fully explored. Similarly, Tu et al. [31] implemented a structured follow-up approach combining telephonic contacts and community health centre visits. This multidisciplinary framework facilitated effective collaboration among healthcare professionals and ensured a seamless transition to post-discharge care.

Kolcu and Ergun [19] emphasised patient education and support through 20 weeks of interventions, including regular assessments and interactions with healthcare providers. This approach fostered adherence, enabled progress monitoring, and allowed for timely interventions. Meanwhile, Sukpattanasrikul et al. [32] incorporated a combination of telephone follow-ups and group meetings, enhancing self-management and providing a sense of community, which further motivated patient participation.

Systematic health assessments, as demonstrated by studies such as those by Sheilini et al. [29] and Kim and Park [30], underline the importance of continuous data collection on vital health indicators. These assessments empower healthcare providers to tailor treatments effectively and engage patients in their health journeys. Overall, regular follow-ups, combined with multidisciplinary support and systematic health assessments, enhance treatment adherence, improve health outcomes, and address potential barriers in hypertensive management strategies.

#### 3.3.5. Theme 5: Environmental Support

The effective management of HTN in older adults benefits significantly from practical assistive devices, environmental modifications, and caregiver involvement. Sheilini et al. [29] highlighted the utility of weekly pill reminder boxes in promoting medication adherence. This intervention proved beneficial by enhancing organisational support for patients and fostering self-reliance. However, its success hinges on the cognitive capabilities of users or supplementary caregiver assistance.

Kolcu and Ergun [19] explored environmental interventions targeting older adults with HTN. Measures such as removing saltshakers and establishing accessible exercise areas led to improved health outcomes. However, the limited scope of their study highlights the need for larger-scale research to evaluate the sustainability and generalizability of these interventions. Sukpattanasrikul et al. [32] emphasised community support and caregiver involvement as critical components in building conducive environments for older adults with chronic illnesses. Their research advocated for fostering reciprocal relationships and integrating family carers into community-based health initiatives. These approaches leveraged social networks to enhance the well-being of older adults with HTN.

Overall, the analysis identified five areas for successful health interventions in community settings: nurse-led empowerment strategies for HTN management, variability in blood pressure outcomes, the importance of tailored education and counselling, the role of regular follow-ups and support, and environmental support. These strategies not only facilitated patient engagement and informed decision-making but also contributed to a supportive environment, ensuring continuity of care. Collectively, these interventions demonstrate the potential to improve health outcomes for older adults with HTN through structured environmental and social support systems.

## 4. Discussion

The management of HTN in older adults remains a pressing public health challenge [36], particularly among older adults [1]. This review highlighted how nurse-led strategies for lifestyle modifications aim at improving HTN among older adults. The review identified five relevant studies that collectively suggest that these strategies are effective in lowering BP levels.

Nurse-led empowerment strategies have emerged as a promising approach to managing HTN in older adults. The literature indicates that these strategies encompass holistic and culturally sensitive interventions, which include tailored education, counselling, and the utilisation of telehealth services [19,29,30,31,32]. Notably, studies have demonstrated significant reductions in systolic blood pressure (SBP) attributable to nurse-led initiatives. For instance, Kost et al. [37] reported a decrease in SBP of 4.41 mm Hg within one month and 6.9 mm Hg over six months, while Bulto et al. [38] noted similar reductions. These findings corroborate the assertion that nurse-led interventions can effectively manage HTN and improve patient outcomes [39,40]. However, the variability in BP reduction across studies suggests that factors such as intervention design, participant demographics, and assessment periods may influence outcomes. Therefore, further research is warranted to validate these findings and explore the underlying mechanisms driving the observed effects.

The successful implementation of nurse-led empowerment strategies hinges on several facilitating factors. Tailored education and counselling emerged as critical components in promoting effective HTN management practices among older adults. The scoping review highlighted that personalised education not only enhances patient engagement but also fosters self-management capabilities [19,29,31]. For instance, studies by Sheilini et al. [29] and Tu et al. [31] demonstrated that customised educational interventions significantly improved self-management outcomes for older adults with HTN. Furthermore, Sukpattanasrikul et al. [32] emphasised the importance of cultural adaptation in educational content to address the specific needs of diverse populations. However, a notable limitation across these studies is the lack of comprehensive detail regarding the implementation processes of personalised education, which constrains the evaluation of their effectiveness and replicability. Future research should aim to delineate specific personalisation methods and assess their relative effectiveness in various contexts.

Regular follow-ups and support mechanisms were identified as essential facilitators for sustaining the adherence to HTN management practices among older adults. Consistent monitoring not only enhances medication compliance but also promotes long-term lifestyle changes necessary for effective BP control [19,29,31,32]. The review noted significant differences in participant selection and support team composition across studies, reflecting varying approaches to addressing patient needs. For example, Tu et al. [31] incorporated community nurses and general practitioners into their support teams for older patients with co-morbid conditions like diabetes, while Sukpattanasrikul et al. [32] included family caregivers as integral members of the support system. These variations highlight the necessity for multidisciplinary approaches tailored to specific patient populations.

Moreover, the existing literature highlights the critical role of follow-up care in achieving better BP control outcomes. Kaur et al. [40] demonstrated that regular follow-ups significantly increased the proportion of patients achieving optimal BP control in a large cohort study conducted in India. Similarly, Peng et al. [41] found that patients attending multiple follow-up appointments exhibited improvements in BP management and self-reported health scores. However, real-world challenges such as patient dropout during long-term follow-ups can hinder the effectiveness of these interventions on a broader scale. Health assessments are another crucial element in managing HTN among older adults. Scheduled evaluations enable healthcare providers to monitor patient progress and adjust treatment plans accordingly [19,29,30]. The variability observed in research findings may stem from differences in the measurement methodologies and participant characteristics across studies. Egan et al. [42] emphasised the importance of routine health check-ups following significant health incidents to optimise HTN management outcomes. Additionally, Ma et al. [43] highlighted that regular assessments can enhance quality of life and reduce mortality rates among frail older individuals with HTN.

Environmental support strategies also play a vital role in facilitating effective HTN management practices among older adults. The scoping review consistently emphasised the significance of environmental modifications to enhance medication adherence and encourage healthy lifestyle choices [19,29,32]. For instance, interventions such as using pill reminder boxes and creating designated areas for physical activity have been shown to improve adherence to prescribed regimens. Furthermore, involving family members in care strategies can foster a supportive environment conducive to lifestyle changes. Cultural considerations are paramount when designing interventions aimed at managing HTN among diverse populations. Research by Yang et al. [44] and Bryant et al. [45] highlights how culturally competent approaches can enhance patient engagement and adherence to treatment protocols within specific communities. Personalised home visits tailored to socioeconomic backgrounds have proven effective in improving health outcomes among economically disadvantaged groups.

In conclusion, while nurse-led empowerment strategies demonstrate considerable potential for managing HTN among older adults, several challenges must be addressed to optimise their effectiveness within community settings. Future research should focus on refining intervention methodologies, standardising implementation processes, and exploring culturally sensitive approaches that resonate with diverse populations. By fostering an integrated model that encompasses education, regular follow-ups, health assessments, and environmental support tailored to individual needs, healthcare providers can significantly improve the quality of life for older adults living with HTN while effectively managing this chronic condition across various community contexts.

### 4.1. Implications for Nursing Practice

The findings of this review indicate the effectiveness of tailored educational interventions and continuous support for managing HTN among older adults. Personalised education, such as culturally adapted interventions and individualised counselling, facilitates self-management, lifestyle modifications, and adherence to treatment plans [29,31,32]. Nurses play a pivotal role in delivering these interventions, emphasising their significance within multidisciplinary teams and community settings. Including family carers, as seen in Sukpattanasrikul et al. [32], highlights the need for adapting strategies to cultural and familial contexts, reinforcing the value of family participation in HTN management.

Additionally, regular follow-ups are vital in enhancing the adherence to medication and sustaining the effectiveness of learned practices. Evidence from studies such as Kaur et al. [40] and Peng et al. [41] demonstrates the positive impact of structured follow-up care on BP control and patient outcomes. However, practical challenges, including patient dropouts during long-term follow-ups and the need for customised approaches in diverse healthcare settings, must be addressed. Policymakers and healthcare professionals should advocate for improved training, resources, and the integration of telehealth and community services to enhance the delivery and sustainability of effective HTN management strategies for older adults.

### 4.2. Strengths and Limitations

The strengths of this review include a comprehensive search strategy that utilised six major databases (CINAHL, MEDLINE, PsycINFO, EMBASE, Web of Science, and Scopus) to ensure a wide coverage of the relevant literature. This review adhered to a rigorous methodology, following established guidelines for scoping reviews, including those from the Joanna Briggs Institute, Arksey and O’Malley, and PRISMA-ScR [20,21,22]. By targeting a specific and underserved population of older adults with HTN, this research has significant clinical and policy implications. The findings are directly applicable to community-based clinical practice, offering a practical roadmap for implementing nurse-led strategies in similar contexts.

Despite these strengths, the study has several limitations that must be acknowledged. First, the review was restricted to English-language literature, which may have excluded relevant research published in other languages or earlier seminal works. This limitation introduces a potential bias toward English-speaking countries and may reduce the generalizability of the findings to diverse geographical or cultural settings. Second, the studies included in the review had their own limitations, particularly concerning sample selection and population scope. Most of the included studies were conducted in specific geographical or cultural contexts and often involved restricted populations, which may limit the applicability of the findings to broader or more diverse populations. Consequently, the insights derived from these studies should be interpreted with caution, as they may not fully represent the experiences and outcomes of other groups. By recognising these strengths and limitations, this review contributes valuable insights into the implementation of nurse-led interventions while highlighting areas for further research to enhance generalizability and inclusivity in future studies.

## 5. Conclusions

This scoping review provides valuable insights into the understanding of nurse-led empowerment strategies for HTN management in older adults within community settings. By synthesising diverse intervention studies, the findings highlight the efficacy of tailored education, structured follow-ups, and environmental modifications in improving blood pressure control, medication adherence, and self-management skills. Insights into personalised care, multidisciplinary support, and cultural sensitivity provide a guide for designing effective, scalable interventions. However, the variability in blood pressure outcomes and study designs highlights the need for standardisation and further investigation. Many interventions demonstrated short-term benefits, but their long-term sustainability and applicability across diverse populations remain underexplored. Challenges such as small sample sizes, geographic limitations, and potential biases also restrict the generalizability of findings. Future research should prioritise longitudinal studies, larger cohorts, and culturally adaptive strategies to enhance global relevance. Ultimately, nurse-led approaches offer a promising path to equitable, community-based HTN care, warranting continued research.

## Figures and Tables

**Table 1:** Search terms (PEO).

**Population**	“older people” OR “elderly” OR “geriatric” OR “seniors” OR “aged”
**Exposure**	“nurse-led” OR “nurse-intervention” And “lifestyle modification”
**Outcome**	“hypertension” OR “blood pressure” OR “systolic and diastolic blood pressure”

**Table 2:** Eligibility criteria.

Inclusion	Exclusion
Older population, aged over 60	Qualitative, systematic reviews (but scan systematic review references for relevant papers)
Nurse-led and lifestyle modification	
HTN blood control	
Only papers published in English language	

**Table 3:** Sample information of included studies.

Authors	Country	Aim of Research	Research Design	Sample	Main Findings
Sheilini et al., 2019 [29]	India	To explore effects of multimodal interventions on medication adherence, quality of life (QoL), HTN, self-efficacy, and blood pressure (BP) outcomes with older people with HTN.	RCT	124 (64 experimental, 60 control) aged 60+ years, Stage I/II HTN, recruited from a clinic. Experimental: Male: 42.2%, Female: 57.8%. Control: Male: 53.3%, Female: 46.7%.	Nurse-led interventions significantly improved medication adherence, HTN knowledge, and self-efficacy, despite no changes in quality of life or blood pressure. In addition, nurses play a crucial role in effectively managing BP.
Kim and Park, 2023 [30]	Korea	To evaluate the impacts of auricular acupressure on BP, stress, and sleep quality in older individuals with essential HTN.	Single-blind RCT	46 females (23 experimental, aged 65–85. 23 control). 100% female. Essential HTN.	Auricular acupressure leads to improvements in sleep quality and reductions in BP and stress levels, thereby serving as a viable nursing intervention for older individuals with HTN.
Tu et al., 2020 [31]	China	To assess the impact of a nurse-led transitional care program on HTN control in older adults with diabetes.	RCT	270 (135 per group), mean age 70.9 years (SD 5.8), aged 60+, 55% male. Uncontrolled HTN.	The nurse-led empowerment strategies programme significantly enhanced health knowledge, increased treatment adherence, and reduced BP. It also reduced hospital readmissions and emergency visits without increasing adverse events.
Kolcu and Ergun, 2020 [19]	Turkey	To examine the impact of a nurse-led HTN management program on QoL, medication adherence, and BP control on older adults.	RCT	76 (37 experimental, 39 control), age ≥65 years. Experimental group: 51.4% male, 48.6% female. Control group: 56.8% male, 43.2% female.	The nurse-led HTN management programme significantly improved medication adherence, QoL, and health knowledge in older adults, and reduce BP, thereby promoting positive lifestyle changes.
Sukpattanasrikul et al., 2021 [32]	Thailand	To assess the impact of a self-management program on self-care, BP, and QoL in older adults with uncontrolled HTN.	Quasi-experimental study	156 (78 experimental, 78 control), age 60+ years, uncontrolled HTN (BP ≥140/90 mmHg). Gender distribution not available.	The self-management programme (SMP) improved self-care, reduced BP, and enhanced QoL in older adults with HTN by incorporating cultural and personalised elements.

**Table 4:** Table of quality appraisal scores.

Study	Quality Appraisal Score
Sheilini et al., 2019 [29]	10
Kim and Park, 2023 [30]	11
Tu et al., 2020 [31]	10
Kolcu and Ergun, 2020 [19]	8
Sukpattanasrikul et al., 2021 [32]	9

## Data Availability

Not applicable.

## Public Involvement Statement

No public involvement in any aspect of this research.

## Guidelines and Standards Statement

This manuscript was drafted against the PRISMA extension for Scoping Reviews (https://www.equator-network.org/reporting-guidelines/prisma-scr/ accessed on 29 September 2024).
